# Modular light sources for microscopy and beyond (ModLight)

**DOI:** 10.1016/j.ohx.2022.e00385

**Published:** 2022-12-12

**Authors:** Graham M. Gibson, Robert Archibald, Mark Main, Akhil Kallepalli

**Affiliations:** a School of Physics and Astronomy, University of Glasgow, Glasgow G12 8QQ, United Kingdom; b James Watt School of Engineering, University of Glasgow, Glasgow G12 8QQ, United Kingdom

**Keywords:** Optics, Photonics, Low cost solutions

## Abstract

Modular light (ModLight) sources can be integrated into complex systems for microscopy, medical imaging, remote sensing, and many more. Motivated by the need for affordable and open-access alternatives that are globally relevant, we have designed and presented light devices that use simple, off-the-shelf components. Red, green, blue, white and near-infrared LEDs are combined using mirrors and X-Cube prisms in novel devices. This modular nature allows portability and mounting flexibility. The ModLight suite can be used with any optical system that requires single- or multi-wavelength illumination such as bright-field and epifluorescence microscopes.

## Graphical abstract

**Figure:**
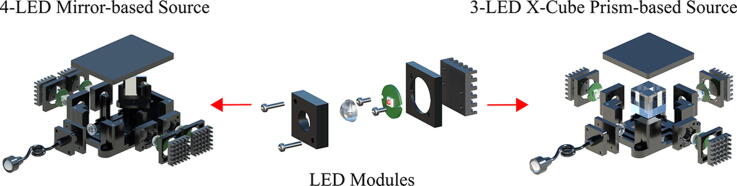

## 1 Specifications table

**Table:** 

**Hardware name**	Modular Light Sources (ModLight)
**Subject area**	• Engineering • Physics • Medical (e.g. pharmaceutical science) • Educational tools and open source alternatives to existing infrastructure
**Hardware type**	Imaging tools
**Closest commercial analog**	No commercial analog is available.
**Open source license**	CC BY-SA 4.0 (Creative Commons Attribution-ShareAlike 4.0 International).
**Cost of hardware**	The cost of two devices is approximately £300 (GBP).
**Source file repository**	Available with the article; https://doi.org/10.5281/zenodo.7385903.
**OSHWA certification UID**	UK000040

## 2 Hardware in context

An imaging system’s fundamental requirement is a source that produces light, which in turn interacts with the object of interest. This interaction results in phenomena such as refraction, absorption, transmission, scattering and reflection. These interactions, quantified by a detector, assist in making inferences regarding the object. The imaging systems are broadly divided into passive (wherein sunlight/ambient light is used to perform remote sensing from, say, satellite-based imaging systems) and active systems (such as fluorescence microscopy where light is shone on the object to image it). To effectively image under specific conditions, special light sources and/or control over multiple sources is necessary. Such control, currently, is achieved by using different LEDs and laser modules through complex programming or switches that are manually operated. Resources are available to illustrate the importance of choosing a light source [1], [2], [3], [4] with a recent focus on sources for specialised imaging with LED sources, such as fluorescence microscopy. There are purpose-built light sources for any application, and new sources continue to be engineered to allow better and more accurate imaging.

New innovations are constantly being presented to the research community, and end users at large. There has been, in recent times, a drive to make research openly accessible and open-source. With the motivation of making light sources more affordable and widely accessible, we introduce two light source designs within the suite of **mod**ular **light** (ModLight) sources that produce collimated light using LEDs.

The ModLight sources use two primary components to direct light, which are:

1. Mirrors; mounted using magnets and easily switchable to reflect light from the LED of choice through the light guide.
2. X-Cube Prisms; which are oriented to allow light from an LED to transmit through the glass material selectively. Originally designed to combine the three channels into white light, we reverse this to transmit light through the light guide.

We designed devices that use simple elements to direct near-infrared, red, green and blue light into a light guide, in this instance. The modular nature of the device designs allows any LED to be used for illuminating an object for imaging. Each device can be made with 3D printing and easily accessible parts. This material is intended to be self-sufficient, to the best of our ability, and we are happy to assist where needed. All the latest files, updates and news from the ModLight suite of tools will constantly be updated on https://kallepallilab.com/modular-microscopy/ and the files (version current at the time of publication) can be accessed at https://doi.org/10.5281/zenodo.7385903.

## 3 Hardware description

The devices made available through this article are specifically designed to facilitate low-cost, robust light sources that can be combined with imaging systems, microscopes, etc. The devices apply the principles of simple optics, along with mirrors and X-Cube prisms to direct light for LEDs into a light guide/fibre. The output of these is combined with a lens, resulting in a collimated beam. The devices are 3D-printed with custom designs to house the LED assembly along with the mirror/X-Cube prism. The devices are custom-built, with novel designs and benefit from a highly modular nature. For instance, a fluorescence microscopy system is usually fitted with a combination of excitation-emission filters and a light source. Applying the technologies presented in this work, the excitation wavelength can be changed with specific LED modules or the white light source from a combination of light using the X-Cube prism can deliver specific wavelengths without needing any adjustment in the imaging setup. Further, these LED modules can be individually built into a suite of sources at specific wavelengths. Should the application need different wavelengths, the LED assemblies can serve as plug-and-play replacements^1^. Concisely,

1. The hardware in this work allows two approaches for delivering collimated light for imaging purposes.
2. White light can be realised as a resulting combination of blue, green and red broadband wavelengths using the X-Cube prism.
3. The modular nature of the LED assemblies allows for changing illuminating wavelengths rapidly and effectively.
4. The custom designs and novel methods of delivering light for an imaging application are accessible and easy to modify. This makes the devices adaptable to almost any imaging setup in their current or modified forms.

## 4 Design files summary

.

**Table:** 

**Design filename**	**File type**	**Open source license**	**Location of the file**	
**Common Parts**				
Fibre Collimator	STL, STEP	CC BY-SA 4.0	Available with the article.	
Fibre Holder	STL, STEP	CC BY-SA 4.0	Available with the article.	
Fibre Mount	STL, STEP	CC BY-SA 4.0	Available with the article.	
Heatsink Drill Guide	STL, STEP	CC BY-SA 4.0	Available with the article.	
Heatsink Mount	STL, STEP	CC BY-SA 4.0	Available with the article.	
PMMA Mount	STL, STEP	CC BY-SA 4.0	Available with the article.	
				
**Mirror-based Light Source**				
MMirror Mount	STL, STEP	CC BY-SA 4.0	Available with the article.	
MLight Box	STL, STEP	CC BY-SA 4.0	Available with the article.	
MLight Box (Lid)	STL, STEP	CC BY-SA 4.0	Available with the article.	
MSource File	OpenSCAD file	CC BY-SA 4.0	Available with the article.	
				
**X-Cube prism-based Light Source**				
XLED Spacer	STL, STEP	CC BY-SA 4.0	Available with the article.	
XLight Mixer	STL, STEP	CC BY-SA 4.0	Available with the article.	
XLight Mixer (Lid)	STL, STEP	CC BY-SA 4.0	Available with the article.	
XSource File	OpenSCAD	CC BY-SA 4.0	Available with the article.	
				
**PCB and LED Control (Electronics)**				
LED Driver (PCB)	PDF, Gerber	CC BY-SA 4.0	Available with the article.	
LED Driver (Schematic)	PDF	CC BY-SA 4.0	Available with the article.	
LED Controller Box	OpenSCAD, STL, STEP	CC BY-SA 4.0	Available with the article.	
Dial	STL	CC BY-SA 4.0	Available with the article.	
Scale Plate	STL	CC BY-SA 4.0	Available with the article.	
**Application - OpenFlexure**				
Condenser Holder	STL	CC BY-SA 4.0	Available with the article.	
Fibre Holder	STL	CC BY-SA 4.0	Available with the article.	

The common components of both light sources include:

1. Fibre Collimator; this part will house the PMMA lens and the fibre, and deliver the collimated beam as output.
2. Fibre Holder; this part holds the fibre against the mount, wherein the PMMA lens is used to collect the light (from the mirror or the X-Cube prism) and focus it into the fibre.
3. Fibre Mount; the part houses the PMMA lens that collects the light from the mirror/X-Cube prism and attaches to the Fibre Holder. Combined, these three parts make up the fibre assembly.
4. Heatsink Drill Guide; The heatsinks (see **Bill of Materials**^2^) are aluminium components that require drill holes to allow for screws. The positioning of these drill holes is directed by the Heatsink Drill Guide. Only one of these will suffice and can be reused for assembly of all the LEDs.
5. Heatsink Mount; this part allows for combining the PMMA Mount with the heatsink to complete the LED assembly.
6. PMMA Mount; this part holds the PMMA lens and has precisely positioned screws to hold the LED assembly together as one unit.

The mirror-based light source requires the mount (**MMirror Mount**) to hold the mirror (**Mirror**^3^) in place. The LED assembly and the mirror mount are all placed in the box, “**MLight Box**”. The source assembly is completed with a lid (**MLight Box (Lid)**). Similarly, the parts for the X-Cube prism-based light source require a compact housing for the LED assemblies and the X-Cube prism placement (**XLight Mixer**). Both lids for the source devices are equipped with magnets to allow for a light-tight yet easy to access build. All components are available and editable through the OpenSCAD files (MSource and XSource Files). The LEDs are controlled using a custom PCB board requiring drivers (**LED-IC**) and potentiometers (**LED-P**); the details of the PCB board and the schematic for the driver are shared in the PDF files, **LED Driver (PCB)** and **LED Driver (Schematic)**.

## 5 Bill of materials

For a detailed bill of materials, please find available a spreadsheet uploaded with this manuscript.

## 6 Build instructions

The 3D printing of all the components takes a few days. In our prototype, we use PLA to test the parts but we recommend printing in Tough PLA for the longevity and robustness of the devices. In both instances, the material is sufficiently capable of dealing with the heat generated by the LEDs for the duration of their operation. We recommend beginning the 3D printing while ordering and awaiting delivery of the various components from the Bill of Materials. The build process for both sources has three common sub-builds:

1. LED assembly (Section 6.1)
2. Fibre-coupled collimated beam delivery (Section 6.2)
3. PCB and Electronics for LED control (Section 6.3)

After the common components have been built, the mirror mount-based (Section 6.4) and/or the X-Cube prism-based device (Section 6.5) can be assembled.

### 6.1 LED Assembly

The LED assembly is modular, and can be completed with any LED at any wavelength, as long as the LED fits within the set-up. Detailed specifications and characteristics of these LEDs are available through the links shared in the Bill of Materials. We envisage that specific applications can be supported by building a suite of LED assemblies, each of which can be replaced/changed in the source device. The heatsinks used in this device are fit-for-purpose. The users can choose to use the LED assembly as is or add thermal paste to dissipate heat from the LEDs. In our experiments, the heatsinks were sufficient.

**Fig. 1:**
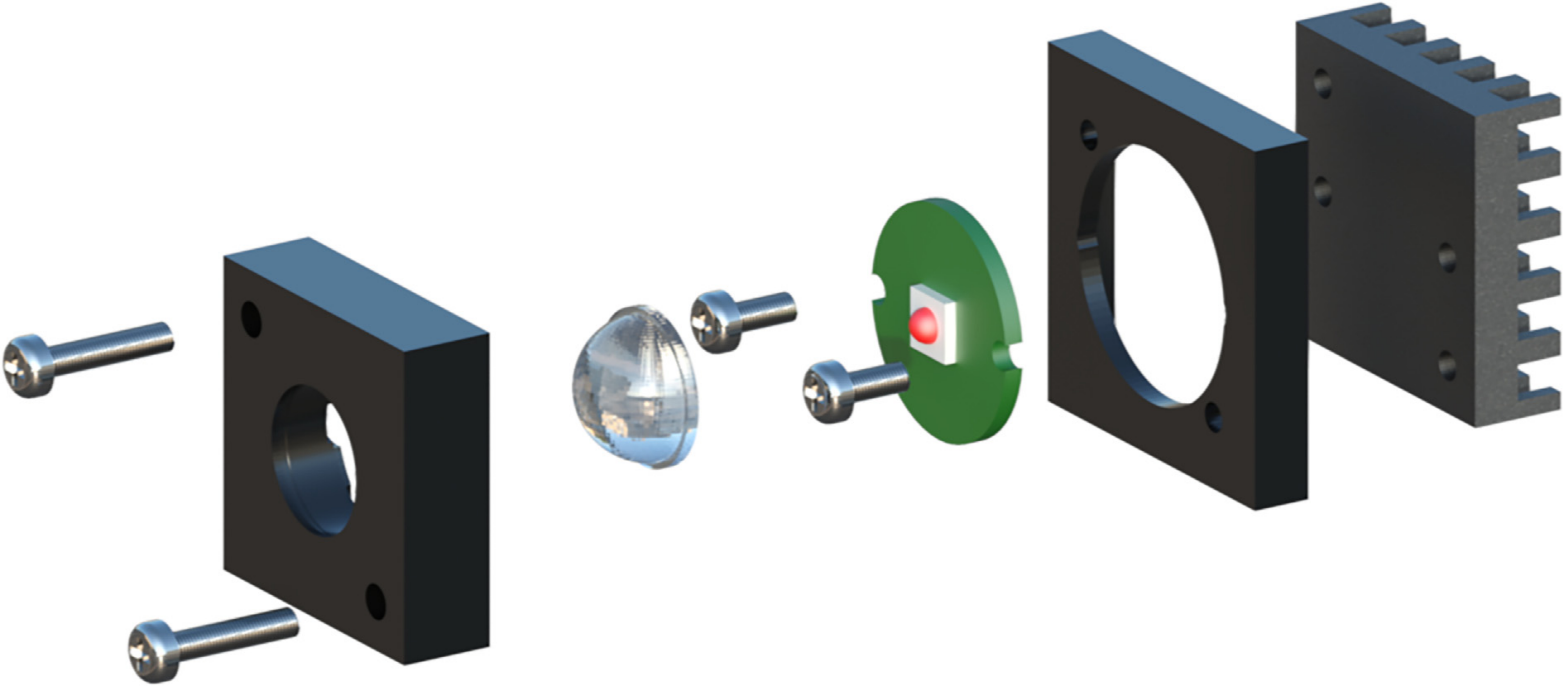
The LED module, common to both sources, can be assembled using 4 screws (2x **S-M2.5–12**, 2x **S-M2.5–6**), the lens mount (**PMMA-f5**), LED modules (**LED-IR-xxx**, **LED-R**, **LED-G**, **LED-B**), a heatsink mount (**Heatsink Mount**) and a heatsink (**HS**). A guide (**Heatsink Drill Guide**) is available for accurate screw hole placements in the heatsink. When using these modules in the ModLight devices, adjusting the screws on the front can be done to optimise the output of the module. allow optimising the output of each module through sub-millimetre level for some corrective steering.

The LED assembly requires 3D-printed parts (**Heatsink Drill Guide**, **Heatsink Mount**, **PMMA mount**) and purchased components (**HS**, **LED-IR-xxx**, **LED-x**, **PMMA-f5**, **S-M2.5–6**, **S-M2.5–12**). The process can be visualised from the illustration, Fig. 1. The LED assembly can be completed through the following steps:

1. Collect the 3D-printed parts. Place the **Heatsink Drill Guide** on top of the heatsink (**HS**) to make screw holes in the precise locations.
2. Once complete, mount the **LED-x** (choice of LED) on top of the heatsink and wire it appropriately using the red and black cables (**EW-R**, **EW-B**). Allow for a sufficient length of cables, depending on your workspace and application.
3. Use the 6 mm M2.5 screws (**S-M2.5–6**) to secure the LED module to the heatsink. Place the **Heatsink Mount** on top to hold the LED and wires in place.
4. Take a 5 mm focal length PMMA lens (**PMMA-f5**) and place it securely inside the **PMMA Mount**. Once completed, align the screw holes and complete the assembly by securing all the components with the 12 mm M2.5 (**S-M2.5–12**) screws.

### 6.2 Fibre-coupled collimated beam delivery

The light that has been directed either by the plane mirror or the X-Cube prism towards the output requires collection and flexible delivery. Further, the output must be a collimated beam for applications such as fluorescence microscopy. Therefore, as described, the common components can be combined to collect and deliver the light to the imaging application/experiment.

**Fig. 2:**
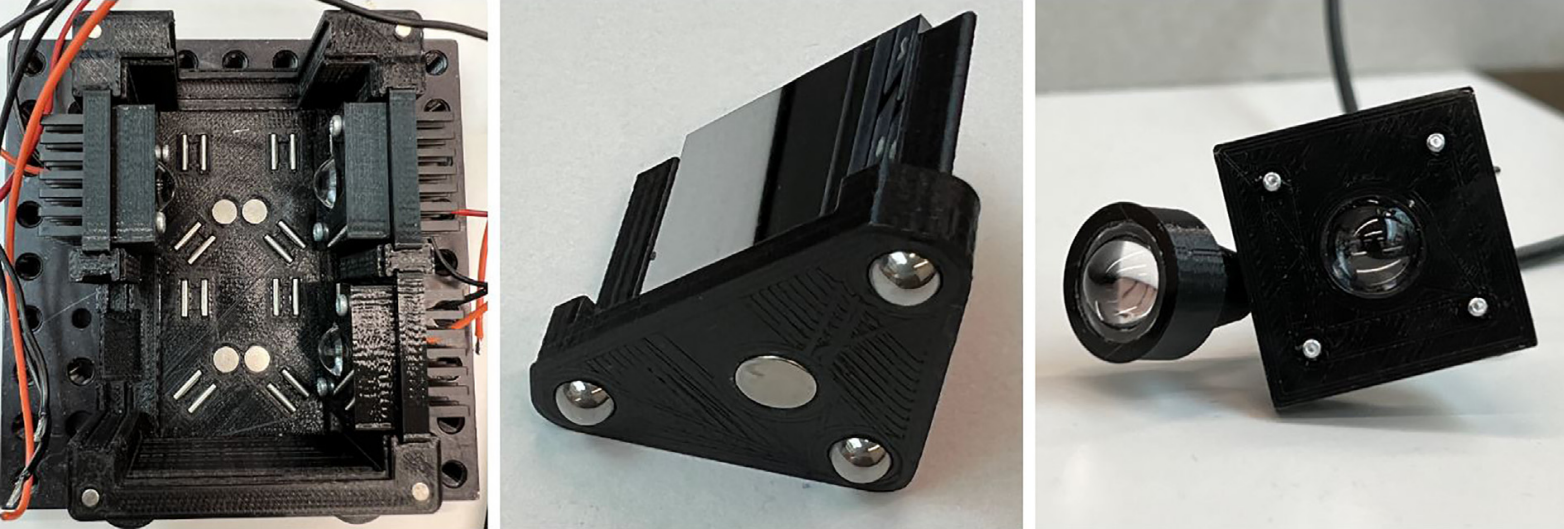
Before assembling all the components of the light source, the base of the light box (*left*), the mirror mount (*middle*) and collimated light delivery through the light guide (*right*) should be prepared. The dowel pins (**P-2x8**) and magnets (**M-6x3**) for the light box; ball bearings (**BB-5 mm**) and magnets (**M-6x3**) for the mirror mount are illustrated, assembled and ready for use. The combination of dowel pins-ball bearings and magnets ensure that the mirror is aligned at 45° to the LED of choice.

1. Collect the 3D-printed parts (**Fibre Mount**, **Fibre Holder**, **Fibre Collimator**) and the purchased components (**PMMA-f5**, **S-M2.5–12**, **Fibre** of a suitable length).
2. Place the 5 mm focal length lens in the **PMMA Mount**. This part of the set up collects the light from the mirror or the X-Cube prism. The mount is then aligned with the **Fibre Holder** which holds one end of the light guide (**Fibre**).
3. The other end of the light guide fits into the **Fibre Collimator**. Before fitting the light guide into the collimator, place the PMMA lens (**PMMA-f5**) on the wider end. Combined with the light guide and the lens, a collimated beam output is realised (Fig. 2 (*right*)).

### 6.3 PCB and Electronics

The electronic circuit schematic for the LED controller is shared in the PDF file **LED Driver (Schematic)**. The circuit is based on the RCD-24–1.00 LED controller module with only a small number of additional components. A PCB board design is shared in the PDF file **LED Driver (PCB)**. The drivers (**LED-IC**) and potentiometers (**LED-P**) are placed on the board, and the LEDs are wired into the controls. This design offers control beyond simply turning the LEDs on and off and is capable of managing intensity outputs.

**Fig. 3:**
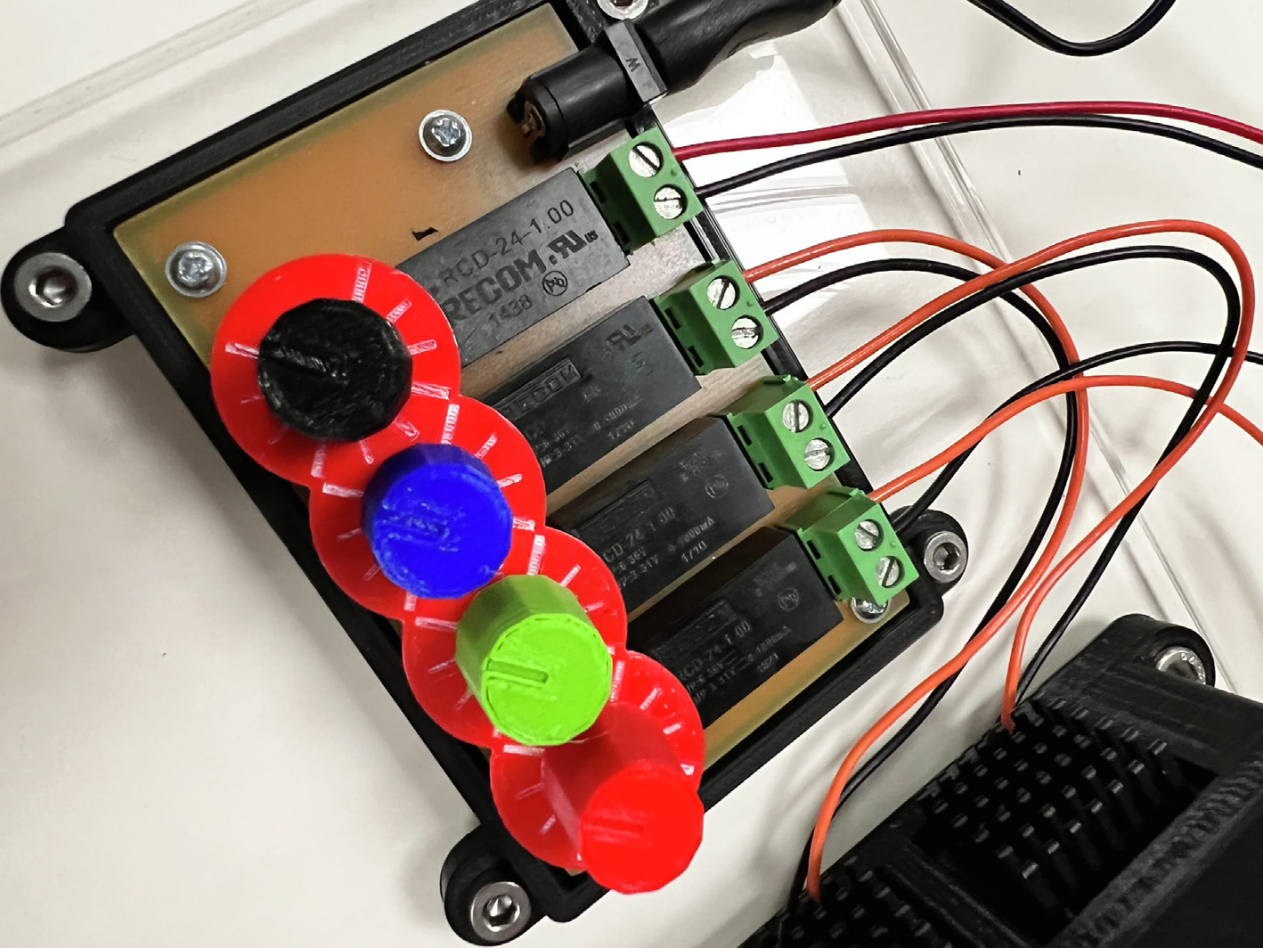
The LEDs are controlled using 4 potentiometers (**LED-P**) and drivers (**LED-IC**). We designed dials, printed with coloured PLA, and placed a scale plate underneath each dial to accurately reproduce the intensity of the LEDs.

All the LEDs have a manufacturer’s maximum rating of 1A. The maximum we output through the driver is 1A (per channel). The power needed to drive the IR LED is the same as the visible LEDs, as per the manufacturer’s guidance. The LED driver is powered by a 12 V 1.5 A power supply, which is capable of powering both devices. The control board is illustrated in Fig. 3, along with individual dials and the scale plate. The LED driver is powered by a 12 V 1.5 A power supply, which is capable of powering both devices.

### 6.4 Devices with Mirrors

**Fig. 4:**
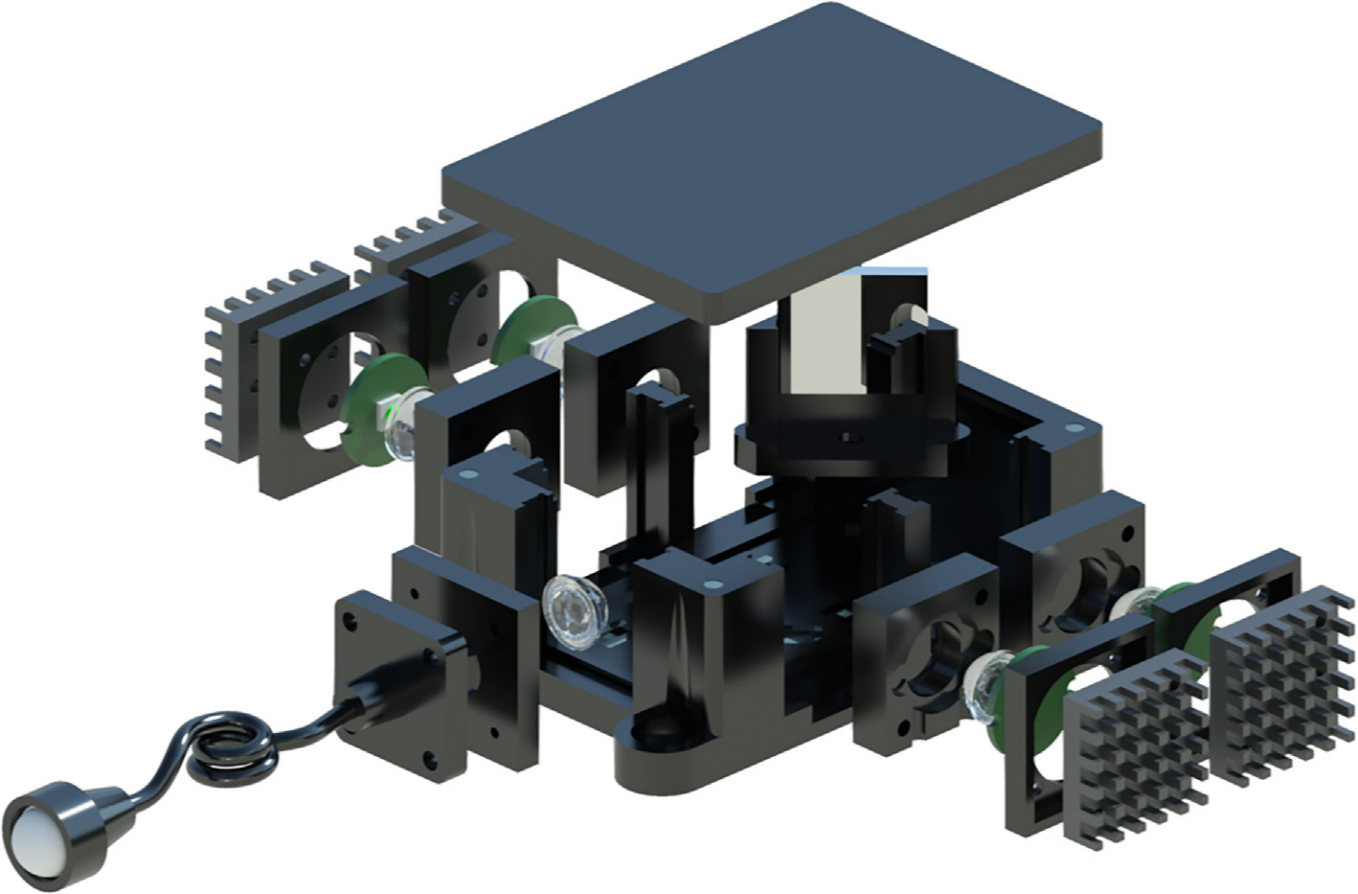
The LED modules can be integrated into the main device using other 3D-printed parts. The components and parts needed are the light box (**MLight Box**) and lid (**MLight Box (Lid)**), magnets (**M-6x3**, **M-3x2**), dowel pins (**P-2x8**), mirror mount (**MMirror Mount** and a mirror (**Mirror**) The ball bearings underneath the mirror mount allow easy place. Once assembled, the light delivery component can be assembled with a combination of lenses (2x **PMMA-f5**), fibre mount (**Fibre Mount**), fibre holder (**Fibre Holder**) and fibre collimator (**Fibre Collimator**). The LED modules are wired to a simple switch that can turn each LED on and off as required.

**Fig. 5:**
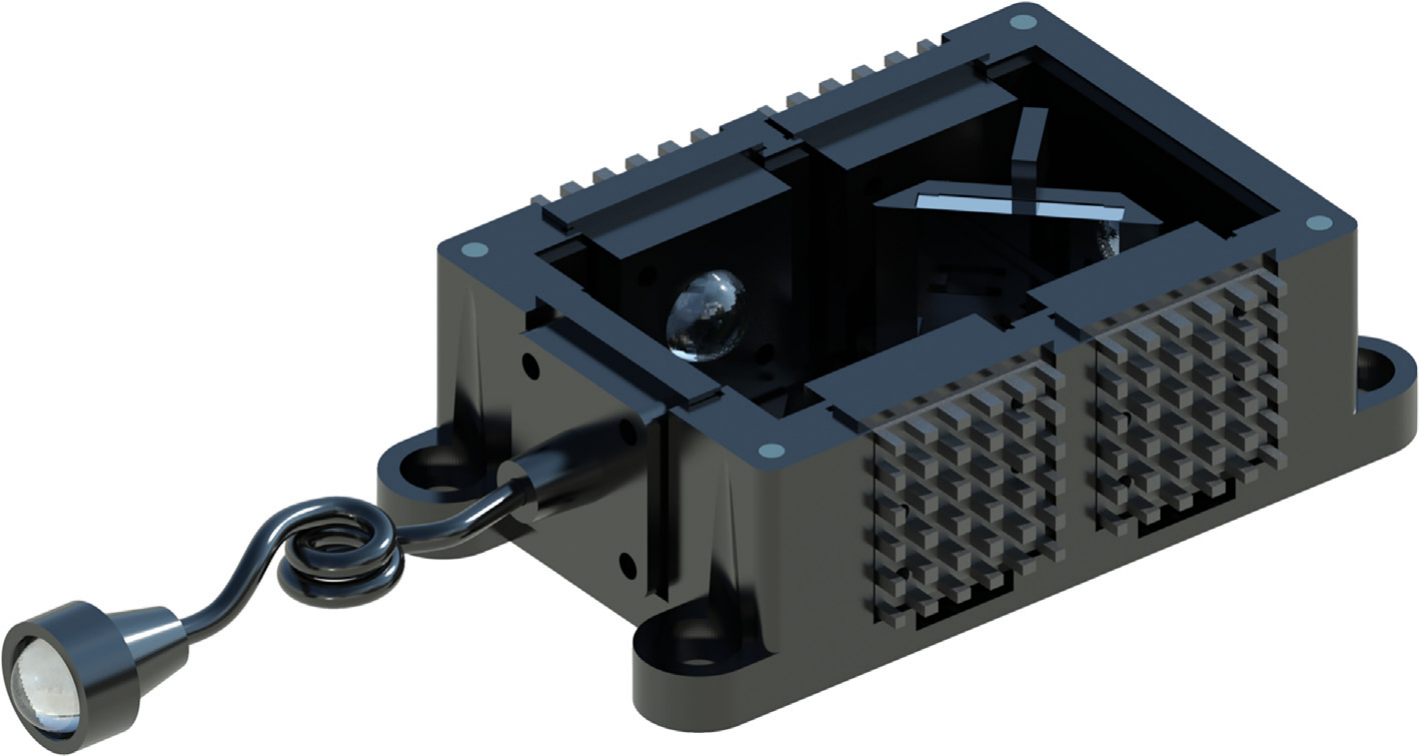
The mirror-mount source allows individual control of LEDs, directing the light from the source to the fibre and through the collimator. The lid of the device can be lifted to move the magnet-supported mount can be into any of the 4 positions corresponding to the LEDs at 45° angles. Once the mirror is in the position of choice, the lid can be replaced for a light-tight source with a collimated beam as output from the collimator.

The LED assemblies can be placed in the slots on the vertical walls of the main housing, **MLight Box**. A detailed illustration of the device is given in the exploded (Fig. 4) and completed views (Fig. 5). The light box can be assembled as follows:

1. Once printed, place the dowel pins (**P-2x8**) in their respective slots on the base of the box.
2. Place and secure the magnets (**M-6x3**) on the base. Ensure that the correct poles of the magnets are aligned by pairing magnets before securing them in place. The four magnets in the base of the box need to be of the opposite polarity as that of the mirror mount.
3. Take the mirror mount (**MMirror Mount**) and secure three ball bearings (**BB-5** **mm**) and a magnet (**M-6x3**) at its base.
4. Place the LED modules in the slots and the mirror (**Mirror**) in the mirror mount in one of the positions to secure it in place.
5. Finally, place four **M-3x2** magnets in the top corners of the light box (**MLight Box**) and four magnets of opposite polarity in the lid, **MLight Box (Lid)**.
6. The fibre coupling assembly described in the previous subsection, *Fibre-couple collimated beam delivery*, can now be placed in the appropriate slot.
7. Place the lid over the box, secured by magnets to complete a light-tight, 4-LED source that can deliver collimated light through the output end of the light guide.

### 6.5 Devices with X-Cubes

**Fig. 6:**
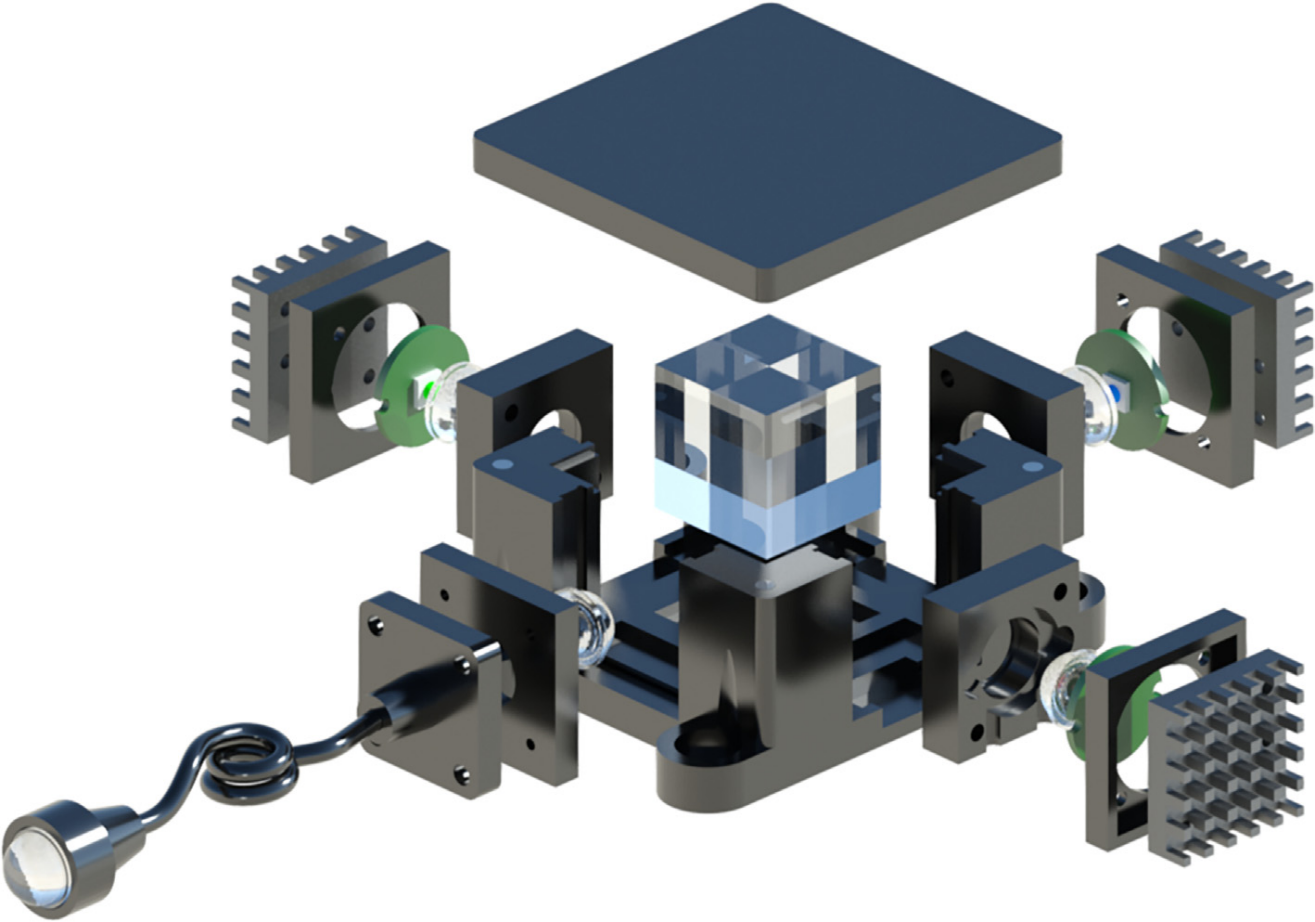
The LED modules can be integrated into the main device using other 3D-printed parts. The components and parts needed are the light box (**XLight Mixer**) and lid (**XLight Mixer (Lid)**), magnets (**M-3x2**) and an X-Cube (**XCube**). Once assembled, the light delivery component can be assembled with the combination of a lenses (2x **PMMA-f5**), fibre mount (**Fibre Mount**), fibre holder (**Fibre Holder**) and fibre collimator (**Fibre Collimator**). The LED modules are wired to a simple switch that can turn each LED on and off as required.

**Fig. 7:**
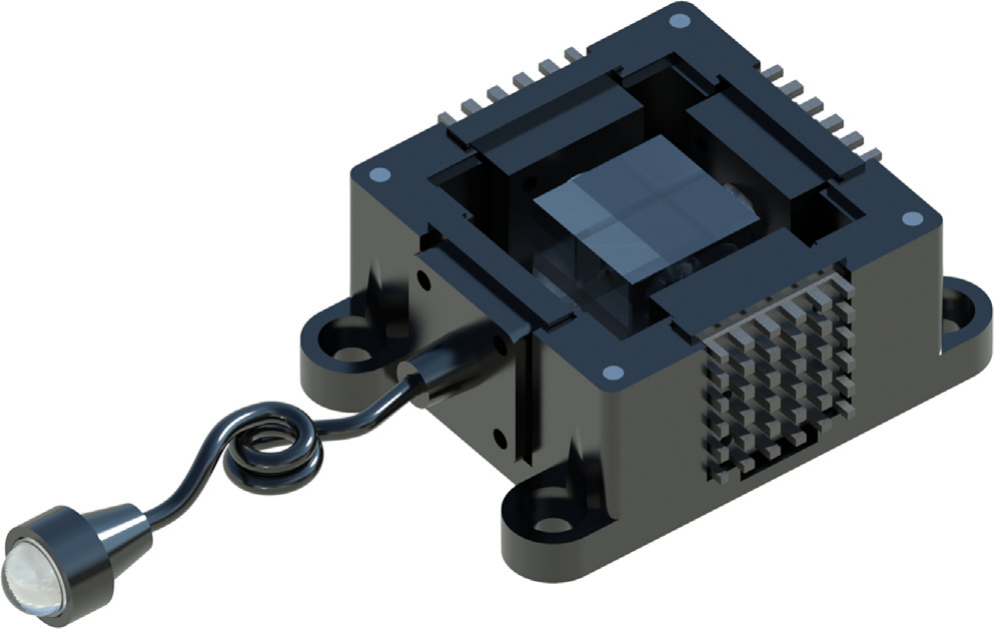
The X-Cube prism-based source allows individual control of LEDs, directing the light from the source to the fibre and through the collimator. This design is significantly more compact than the mirror-based source, and allows for compatibility with fluorescence microscopy systems that integrate multiple light sources and filter cube combinations, without needing to change sources. This single source can combine multiple broadband sources simultaneously, and is capable of delivering appropriate excitation wavelengths to the sample. Once the X-Cube prism is in place, the lid can be replaced for a light-tight source with a collimated beam as output from the collimator.

The design of the X-Cube prism-based source allows for a much more compact design and simultaneous operation of multiple wavelengths. Either individual LEDs can be used for a “pure” output or multiple wavelengths can be combined into a single source and the output can be filtered in the experiment; the choice is available to the user. A detailed illustration of the device is given in the exploded (Fig. 6) and completed views (Fig. 7). The device can be assembled in the following steps:

1. Once printed, secure the X-Cube prism (**Xcube**) in the correct position, aligning the LEDs appropriately to the correct sides of the prism.
2. Place the LED modules in the slots.
3. Finally, place four **M-3x2** magnets in the top corners of the light box (**XLight Mixer**) and four magnets of opposite polarity in the lid, **XLight Mixer (Lid)**.
4. The fibre coupling assembly described in the previous subsection, *Fibre-couple collimated beam delivery*, can now be placed in the appropriate slot.
5. Place the lid over the box, secured by magnets to complete a light-tight, 3-LED source that can deliver collimated light through the output end of the light guide.

## 7 Operation instructions

The devices are designed to operate in a safe and simple manner. Once assembled, the devices can be operated safely under any conditions. The detailed set up and operating instructions are shared through a video (“**OperatingInstructions**”) available with the article.

## 8 Validation and characterisation

Both devices use optical components and 3D-printed housing to achieve light manipulation and deliver a collimated beam of light. They can be used in any application where such an illumination strategy is needed. Further, the intensity of the beam can also be controlled. The devices in operation are shown through the video (“**OperationValidationCharacterisation**”) made available with this article. The capabilities of both devices are similar as the objective is to generate a collimated output. They are only differentiated by access to the primary optical components: X-Cube Prisms and Mirrors. One of the key drawbacks of the X-Cube prism is its inefficiency in near-infrared wavelengths. This challenge is overcome in the mirror-based design as off-the-shelf silver/polished mirrors are capable of reflecting near-infrared wavelengths.

### 8.1 LED Characteristics

**Fig. 8:**
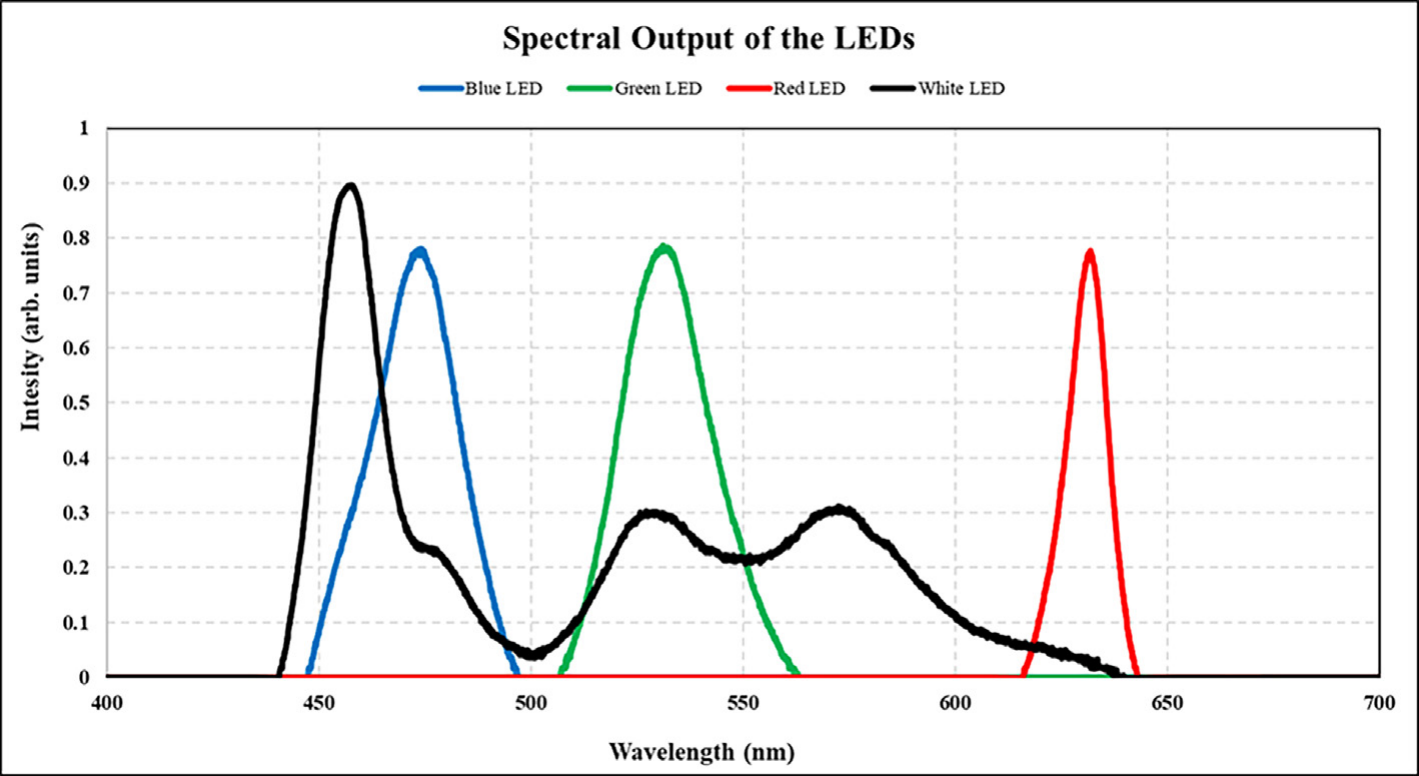
The optical output of the LEDs is quantified through power and spectral measurements. The spectral measurements are illustrated here. The power output of the red, green and blue LEDs is measured using a power meter (CSS100, Thorlabs, Inc.). Note that white light illumination when using the X-Cube prism is a sum total of the three outputs from the single-channel LEDs.

LEDs are usually characterised by their power and spectral properties. The output power was found to be as described in the manufacturer’s technical specifications (as given in links accessible through the Bill of Materials). Using a power meter (PM100D coupled with a S130VC detector, Thorlabs, Inc.), the output of the LEDs was verified in comparison to the manufacturer’s specification sheets. The spectral output was also verified using a spectrometer (CSS100, Thorlabs, Inc.). The resulting spectra from the broadband and white LEDs is given in Fig. 8.

### 8.2 Application Note - Microscopy

**Fig. 9:**
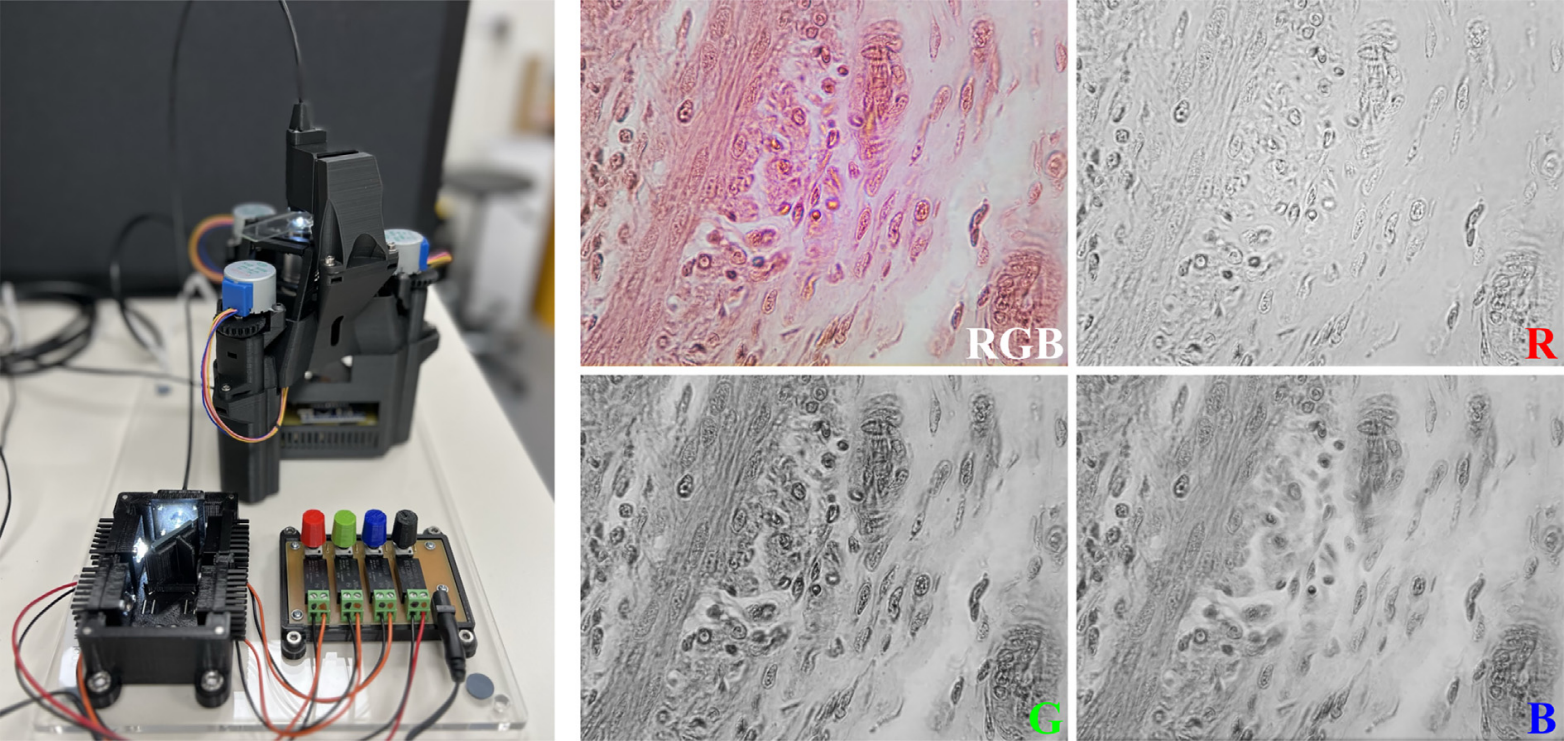
The ModLight device (using a mirror) is equipped with LED-R, LED-G, LED-B and LED-W (spectral curves in Fig. 8). The mirror is moved to each LED and the light is directed through the light guide to the sample in the microscope (Dog Stomach Secretory). Correspondingly, the images captured are shown when illuminated by white (**RGB**), red (**R**), green (**G**) and blue (**B**) light.

**Fig. 10:**
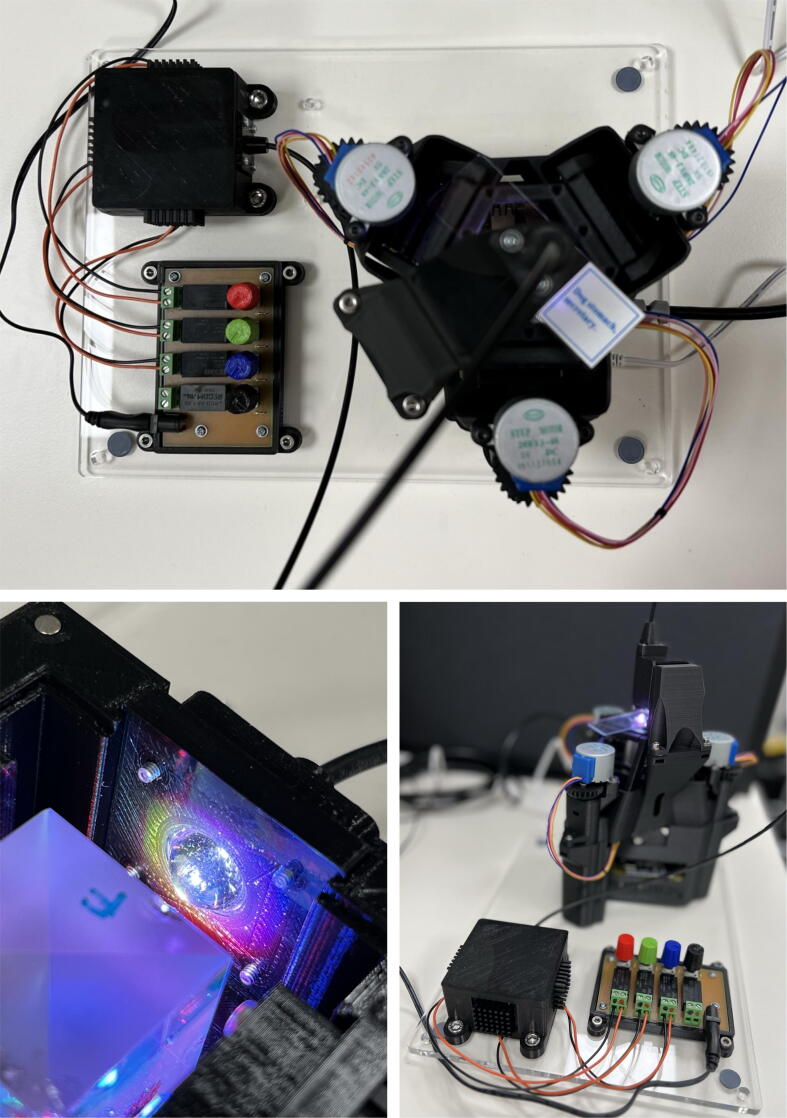
The ModLight device (using an X-Cube prism) is coupled here with an OpenFlexure Delta Stage microscope (**top**). The misalignment of colours (**bottom-left**) can be seen, but is optimised to ensure that the colours overlap for combined white light over the approximate region of the collection lens’ diameter by adjusting the LED assembly. The collimated beam output is used to image a biological sample (Dog Stomach Secretory, **bottom-right**).

A simple application of the ModLight device is shared here, with the OpenFlexure microscope (https://openflexure.org/). The Delta stage, available through the OpenFlexure project, achieves high resolution and stability when imaging samples. The ModLight device allows illumination control by mixing multiple wavelengths (using the X-Cube prism, Figure 9) or with four LEDs operating one at a time (using the mirror-based device, Fig. 10).

The Delta Stage (OpenFlexure project) can move the sample in three dimensions with a mechanism of flexures, similar to the standard microscope, but with static optics and a high degree of stability. We introduced minor modifications (**Condenser**_**Holder**_**Modified** and **Fibre**_**Holder**_**Modified**) to accommodate the light guide, replacing the LED from the original design. In both set ups using the ModLight devices, we successful illustrate broadband imaging either using specific wavelengths of white light LEDs.

In summary,

1. Effective, flexible, modular and low-cost illumination capabilities in the visible and near-infrared wavelengths are achieved.
2. Multiple LED modules can be built and used in both systems without needing completely new devices at each instance. While the LED modules can be used directly with a microscope as an illumination module, there are certain advantages and disadvantages to this approach. While the advantage is simplicity, the disadvantages of existing systems are that each module will have to be individually powered and changed when the illumination strategy changes. This is a manual process which requires multiple rewiring steps and physical interchanging of LEDs. Alternatively, multiple power supplies have to be wired with each LED, still requiring the user to change the modules. In comparison, using ModLight devices allows stability of one output through the light pipe, multiple LEDs ready to use and a single step of wiring. In the end, the choice remains with the end user.
3. Individual LED modules and their output intensity can be controlled using the electronics modules shared (Section 6.3).
4. Using the X-Cube Prism-based design, multiple wavelengths (in the visible region) can be mixed to realise a multi-wavelength system.
5. Using the mirror-based devices, multiple wavelengths in the visible and near-infrared spectrum can be simultaneously set up. The quick-release magnets of the light tight box and the mirror mount ensure simple, safe and rapid switching between the 4 options.
6. Both devices can be operated for a long duration. Within our laboratory testing, the devices have performed without any complications. The combination of low-cost LEDs and heatsinks ensures quick and inexpensive replacement of malfuncting LEDs, and the thermal output of the system is effectively dissipated, respectively.

The limitations of these devices detailed below:

1. The mirror-based device is limited in operating beyond the near-infrared due to the nature of a polished mirror.
  This challenge can be overcome by using other mirrors that can reflect longer wavelengths, available from Thorlabs, Ltd. and Comar Optics.
2. The mirror-based device does not allow mixing multiple wavelengths due to the single mirror and LED combinations that can be achieved at a time.
  This limitation can be overcome by using the X-Cube prism device.
3. Both devices can operate efficiently in the visible region of the spectrum. However, for near-infrared illumination, the X-Cube prism-based design is not suitable.
  We recommend using the mirror-based device for a broader capability in visible and infrared wavelengths (up to 20 μm).

## CRediT authorship contribution statement

**Graham M Gibson:** Conceptualization, Methodology, Validation, Resources, Data curation, Writing - review & editing. **Robert Archibald:** Methodology, Validation. **Mark Main:** Visualization, Writing - review & editing. **Akhil Kallepalli:** Methodology, Validation, Investigation, Writing - original draft, Writing - review & editing.

## Declaration of Competing Interest

The authors declare that they have no known competing financial interests or personal relationships that could have appeared to influence the work reported in this paper.

## Footnotes

1 All the components from the *Bill of Materials* and 3D-printed components from the *Design files summary* are mentioned in **bold** in the text.

3 See Bill of Materials, available with the article as a separate spreadsheet.

## Footnotes

2 Available with the article as a separate spreadsheet.
